# Prognosis in Insanity

**Published:** 1898-07-23

**Authors:** F. St. J. Bullen

**Affiliations:** late Medical Officer West Riding Asylum, Wakefield


### Medical Progress and Hospital Clinics.

[*The Editor will be glad to receive offers of co-operation and contributions from members of the profession. All letters should be addressed to* The Editor, at the Office, 28 & 29, Southampton Street, Strand, London, W.C.]

### PROGNOSIS IN INSANITY.

By F. St. J. Bullen, M.R.C.S., late Medical Officer West Riding Asylum, Wakefield.

It would not be possible within the limits of this article to enter fully into the numerous considerations which have to be weighed in forming a prognosis in insanity, much less to treat of that of different varieties of insanity in detail. I have here endeavoured briefly to set forth some general rules which will serve for guidance, and at the same time have only attempted to deal with one aspect of the subject, viz., the circumstances indicating an unfavourable outlook. These rules will be dealt with under the following headings. (1) Duration of Mental Symptoms. Any unnecessary delay in commencing treatment. It is abundantly proved by statistics that the longer the duration of the mental disorder previous to treatment the more unfavourable the outlook, and that such is to be expected is readily apparent. But this long duration also implies, in a considerable number of cases, that the insanity is of an insidious, slowly-developing form, and therefore less curable; hence, from both considerations, the factor of time is important. But an exception must be made in the case of recurrent insanities, in which even a year's duration of symptoms allows of a ready recovery. The time element will receive further short allusion in discussing some more important varieties of mental disorder. (2) Age: Youth is favourable to recovery, unless there be present an overpowering hereditary taint, or a complication such as phthisis, long-continued masturbation, &c. Advancing years decrease the hope of recovery until senility be reached, when, apart from certain physical complications the prognosis somewhat improves. An exception should be made in the case of climacteric insanity from æt. 45 to 50. This, however, is less favourable when occurring after 50 years of age than when attacking earlier, and so follows the general rule. The period from 30 to 50 has its own grave risk in general paralysis, especially when there have been no previous attacks of insanity in the individual. (3) Heredity: Put briefly, its influence, if strong, endangers an early breakdown and, perhaps, incurability or frequent relapses. If weak, early breakdown and rapid recovery or insanity only developed at a later period in life. Thus adolescent insanity has always a serious aspect, though a large recovery list, because it implies a degenerate stock. Even though a first attack be not lasting, yet the danger of relapses, of some degree of permanent mental and moral change (short of insanity), is considerable. When heredity is strongly marked, parental or ancestral insanity having existed, there is in breakdown at adolescence a probability that permanent mental disorder will ensue or frequent relapses, which, if not checked before mid-life end in premature mental enfeeblement. In puerperal, lactational, and climacteric cases its influence is again seen to be unfavourable; in the first increasing the chance of recurrence at subsequent child-births and in the two last retarding or endangering recovery. (4) Recurrence may be suitably classed with the preceding, implying as it generally does hereditary instability. The prognosis is bad from the first when the breakdown occurs in youth, and with each relapse becomes worse. So it is when the exciting cause may be recurrent as with child-birth, lactation, alcoholic excess. But as regards temporary recovery, the prognosis is not unfavourable; in fact, a large proportion of recurrent cases quickly regain their (relatively) normal state in the earlier attacks. (5) Causation of the insanity: The relative importance and nature of the presumed cause need reflection. On the whole, it may be said that the less adequate the cause appears, the worse the prognosis, inasmuch as thereby predisposition is implied. Such causes, predisposing, exciting, or both, which are really potential, *e.g.*, adolescence, climacteric, senility, child-birth, alcoholic excess, acute anxiety, severe hardship, prolonged stress, exhausting maladies, those in short which disturb even normal equilibrium, more satisfactorily account for breakdown, that is provided the patient has not too readily succumbed. When the causes appear insufficient, or have acted with undue promptitude, or on the other hand the cause, be it slight or grave, has been long acting and moreover productive of physical deterioration, *e.g.*, syphilis, alcoholism, traumatic lesion, visceral and circulatory diseases, the prognosis must be unsatisfactory. (6) Periodicity in attacks: This is always an unfavourable indication, whether it shows itself in more or less regular exacerbations of the current disorder, or in successive attacks with intervals of calm, or in alternating states of excitement and depression. The various forms of established "Folie circulaire," so far as recovery is concerned, are well-nigh hopeless, although dementia is often long postponed. Thus periodicity, when appearing in youth with a neurotic history, must be regarded as decidedly unfavourable, and in later life even lengthy intervals do not contradict this, provided this feature is observable. (7) Acuteness of attack. The prognosis will be influenced by the degree of severity of mental disorder, and by the physical ability to withstand the shock of this. In moderately acute cases the outlook is in great proportion only unfavourable should hereditary taint be very strongly marked, the cause long in operation and of a nature to also produce permanent physical disease, the patient between æt. 30 and 50, or should some other unfavourable conditions exist such as have been or will be mentioned.

Acute and delirious forms of mania and melancholia are very dangerous both to life and mental recovery. The outlook is rendered more ominous the older the patient, the more the constitution be impaired by intemperate or vicious habits; by physical disease; persistent refusal of food; by pyrexia above 100 deg. in mania, reaching 100 deg. in melancholia; rapid pulse, prolonged insomnia, and the typhoidal state. In cases where no marked heredity exists, where the breakdown is the apparent result of long years of mental stress or hard living, and the age is favourable to the occurrence of general paralysis; acute delirious mania is to be regarded with great anxiety, as being especially fatal.

A bad prognosis need not be given in cases of Mania Transitoria, as recovery usually ensues and the attack seldom recurs. The termination of acute melancholia is always rendered more grave by the possibility of suicide. Taking a large number of melancholic cases, under asylum treatment, suicidal inclination is found in over 60 per cent., though the intensity varies considerably in different forms; over 30 per cent. of all are actively suicidal. The duration of an acute attack of either mania or melancholia influences the prognosis, especially in the former case; and even sub-acute or remittent symptoms, if persisting over a year, threaten to become chronic.

About one-fifth of all cases of mania become mentally crippled; melancholia is more recoverable from.

(*To be continued.*)

